# Understanding the relationships between mental disorders, self-reported health outcomes and positive mental health: findings from a national survey

**DOI:** 10.1186/s12955-020-01308-0

**Published:** 2020-03-04

**Authors:** Janhavi Ajit Vaingankar, Siow Ann Chong, Edimansyah Abdin, Fiona Devi Siva Kumar, Boon Yiang Chua, Rajeswari Sambasivam, Saleha Shafie, Anitha Jeyagurunathan, Esmond Seow, Mythily Subramaniam

**Affiliations:** Research Division, Institute of Mental Health, 10 Buangkok View, Singapore, 539747 Singapore

**Keywords:** Mental well-being, Mood disorders, Anxiety, Mediation, Quality of life

## Abstract

**Background:**

The link between mental illness and mental health is gaining focus in research and practice. This study aimed to investigate the association of mental disorders with positive mental health (PMH), overall health and quality of life. In addition, the role of PMH in mediating the relationships between mental disorders and health outcomes was assessed.

**Methods:**

The study sample comprised 2270 residents aged 18 years and above who participated in a nationally representative, cross-sectional survey estimating the lifetime prevalence of mood, anxiety and alcohol use disorders, and health outcomes (self-reported overall health, quality of life and PMH) in Singapore. The Positive Mental Health Instrument was used to estimate the level of Total PMH among the respondents with and without mental disorders. Associations between mental disorders and health outcomes were assessed through regression models. Path analyses were conducted to investigate mediating role of PMH.

**Results:**

Total PMH (Mean ± SD) was significantly lower among individuals having any of the studied lifetime mental disorders (4.23 ± 0.64 versus 4.50 ± 0.67 among those without these disorders). Although having a mood or anxiety disorder was associated with significantly lower Total PMH even after controlling for socio-demographic characteristics, similar relationship was not observed for alcohol use disorders. History of any mental disorders was also associated with lower overall health and quality of life. Total PMH mediated the relationships between mental disorders and overall health and quality of life by reducing the effect sizes for the associations between mental disorders and these health outcomes.

**Conclusions:**

Mental disorders were associated with poor health outcomes in affected individuals. This study showed that PMH can mediate the relationships between mental disorders and health outcomes, and act as an underlying mechanism to improve overall health and quality of life in individuals with mental disorders. Findings thus highlight the significance of incorporating mental health promotion and interventions in clinical populations.

## Background

Singapore is a Southeast Asian country with a multi-ethnic resident (Citizens and Permanent Residents) population of 3.96 million comprising 74.3% Chinese, 13.4% Malay, 9% Indians and other ethnic groups (3.3%) [1]. Since 2007, there has been a concerted effort to assess the state of mental health of the population. Two large, national, cross-sectional, mental health surveys – Singapore Mental Health Study (SMHS) 2010 and 2016 - have provided important estimates on the prevalence and correlates of key mental disorders in Singapore; one in seven individuals in the population (13.9%) were found to have at least one mood, anxiety or alcohol use disorder in their lifetimes [2, 3].

Mental disorders have a profound impact on the lives of those affected in terms of disability [4], morbidity and mortality [5], and quality of life [6]. Globally, the prevalence of mental disorders is also on the rise. The pooled lifetime prevalence of common mental disorders from 85 surveys conducted between 1980 and 2013 across 39 countries was found to be 29.2% [7]. Mental, neurological and substance use disorders and associated self-harm were recently shown to contribute to 19% of the total disability-adjusted life years based on ecological studies conducted in the Americas [8]. This is much higher than the previously reported global figure of 10.4% [4], 11.5% in Singapore [9] and 6.2% in Korea [10]. These trends indicate that despite the advancements in mental healthcare services and effectiveness of therapeutic interventions, prevention and management of mental disorders remains an uphill task. There is growing interest and evidence on the public health benefits of mental health improvement through mental health promotion.

Mental health is the capacity to realise one’s potential, cope with life’s stresses, work productively and contribute to the community [11]. This definition has its foundation in positive mental health (PMH), a construct first proposed by Jahoda [12] who described PMH as individuals’ attitudes towards themselves and the environment and their ability to adapt to situations. Keyes’s two continua model [13] of PMH proposes that mental illness and health are distinct yet correlated constructs that are linked by a continuum which are complementary to each other. By virtue of this belief, persons with mental disorders can at the same time experience positive emotions, form close relationships, have a purposeful life and function well, all of which relate to PMH. This state of ‘flourishing’ is deemed important to ensure higher functioning and better outcomes in individuals. The model has been used to assess the level of flourishing and complete mental health (defined as absence of mood, anxiety or substance use disorder) in a Canadian general population sample [14]. Research has also shown that the combined effect of mental distress and PMH can better predict psychosocial functioning than their independent effects [13]. Following research in the field of mental wellbeing over the past two decades, the literature linking mental disorders with components of mental well-being has increased. Higher levels of PMH are also found to be associated with positive outcomes such as lesser suicidal ideation [15], symptom severity [16, 17], and better remission [17]. This is clearly an indication that PMH can interact and transform the effect of mental disorders and related health outcomes.

However, research in this area is still limited, specifically in relation to alcohol use which has largely been investigated in adolescent populations. Furthermore, most of the previous research has also originated or focused on Western populations. It is believed that group norms and behaviours in relation to mental health and treatment are influenced by social constructs such as race, religion and language [18, 19]. Moreover, it is argued that mental well-being theories are influenced by an ‘individualistic’ notion of well-being that are more suited to Western societies [20] and believed to be more consistent in more assertive individuals with clear self-knowledge [21]. Therefore, there is a need for culturally specific and relevant investigation into mental well-being among Asian populations.

As part of the SMHS 2010 conducted in Singapore, a tool to measure PMH was developed using mixed methods [22, 23]. The development was guided by a narrative literature review, supplemented by focus group discussions among Singapore residents and validated through a series of psychometric analyses. The conceptual underpinnings for this development were grounded in theories of psychological (eudemonic) and subjective (hedonic) well-being where PMH was regarded as a multi-dimensional construct relating to a person’s interpersonal skills, emotional support and coping strategies. It also encompassed their religious and spiritual awareness, goals, autonomy and affective state of mind. Studies in the area of PMH have since yielded information on its sociodemographic correlates in the Singapore’s general population [24, 25] and in people with mental disorders [26]. The later study also found that PMH varied by gender in people with schizophrenia [27] and showed that PMH was positively associated with life satisfaction and general functioning, but negatively related to depressive symptom severity in mental health service users [16].

The earlier studies were, however, conducted in non-representative and comparatively smaller samples, and limited in the type of information available on mental disorders and health related outcomes such as quality of life. Moreover, examining the role of PMH in mediating the relationship between mental disorders and health outcomes heightens our understanding of how quality of life of people with mental disorders could be influenced positively. Using data from the SMHS 2016, this study aimed to estimate the level of PMH among those with and without lifetime mental disorders, and assess the relationships between mental disorders, health outcomes (self-rated overall health, quality of life) and PMH. Additionally, the study investigated the mediating role of PMH.

## Methods

The SMHS 2016 was a national cross-sectional household survey conducted between August 2016 and April 2018. Ethical approval was obtained from the National Healthcare Group’s Domain Specific Review Board prior to start of the study. Written informed consent was obtained from all respondents and from parents or legal guardians of those aged below 21 years. The study details have been published in a previous article [3]. Briefly, a person-level sample was derived randomly using disproportionate stratified sampling from a national database of all Singapore residents aged 18 years and over. A face-to-face household survey was conducted, and attrition data were obtained from all residents. Participants were Singapore Residents (Citizens and Permanent Residents) aged 18 years and over, residing in Singapore at the time of the survey and able to understand the survey in English, Chinese or Malay language. Residents who were institutionalized, uncontactable due to sampling frame errors or unable to be interviewed due to severe physical or mental health problems or language ineligibility were excluded from the survey.

A total of 6126 residents participated in the survey giving a response rate of 69.5%. Of these, 4916 respondents who were English literate were invited to complete the self-administered Positive Mental Health Instrument. They were provided with the questionnaire in a postage paid sealable envelope and asked to mail the completed questionnaire back to the study team. This process allowed privacy, adequate time and minimised social desirability bias for the respondents. A total of 2337 respondents returned the completed questionnaires; of these, 67 questionnaires were excluded due to pattern answers or missing data. Data from the remaining 2270 respondents were used in this analysis, yielding an overall response rate of 30%.

### Sociodemographic information

All respondents completed a sociodemographic questionnaire that obtained information on their age, gender, ethnicity, marital status, education, employment and monthly household income.

### Health measures and outcomes

#### Positive mental health

A self-administered 47-item Positive Mental Health Instrument (PMHI) was used to assess PMH. It covers six dimensions: general coping, emotional support, spirituality, interpersonal skills, personal growth and autonomy, and global affect which was developed and validated after extensive qualitative and quantitative investigations among the multi-ethnic adult population in Singapore [22, 23]. For the first five subscales, respondents are asked how much the items describe them on a scale from ‘not at all like me’ to ‘exactly like me’. The ‘global affect’ subscale includes a list of five affect indicators and requires users to indicate ‘how often over the past four weeks they felt – calm, peaceful, etc’. using a 5-point response scale from ‘never or very rarely’ to ‘very often or always’. All items have a weight of 1; total PMH score was obtained by adding scores of all items and dividing by 47. Cronbach’s alpha (α) for the PMHI was found to be 0.951 in the study sample (α = 0.942 and α = 0.951 among those with and without any lifetime mental disorders, respectively). In an earlier study, total PMH norm score of 4.61 (SD = 0.8) was established in Singapore [25].

#### Mental disorders

The Composite International Diagnostic Interview version 3.0 (CIDI 3.0) [28] was administered to the respondents by trained interviewers. The CIDI is a fully structured instrument where interviewees were first administered a screener questionnaire that asks them about ever experiencing symptoms of mental disorders such as major depressive disorder (MDD), bipolar disorder, etc. Those who endorsed any of these symptoms were then administered detailed disorder-specific modules. Using the Diagnostic and Statistical Manual of Mental Disorders, 4th Edition (DSM-IV) diagnostic algorithms with hierarchy rules, psychiatric diagnoses of lifetime mental disorders were obtained. The CIDI diagnosed lifetime prevalence estimates of mood, anxiety and substance use disorders have demonstrated high level of concordance with clinical measures such as the Structured Clinical Interview for DSM-IV with a receiver operating curve area of 0.76 [29]. Disorders included in the SMHS 2016 were MDD, bipolar disorder, generalised anxiety disorder (GAD), obsessive compulsive disorder (OCD) and alcohol use disorders (AUD) (alcohol abuse and/or dependence). Mention of ‘any mental disorder’ in the subsequent sections of the manuscript refers to the diagnosis of at least one of these five conditions covered in the SMHS 2016, namely, MDD, bipolar disorder, GAD, OCD and AUD.

#### Chronic physical conditions

Respondents reported being ever diagnosed by a doctor with a chronic physical condition from a list of common conditions considered prevalent in Singapore’s population. These were respiratory disorders such as asthma, chronic lung disease including chronic bronchitis or emphysema, diabetes, hypertension, hyperlipidemia, chronic pain (arthritis or rheumatism, back problems including disk or spine, migraine headaches), cancers, neurological disorders (epilepsy, convulsion, Parkinson’s disease), cardiovascular disorders (stroke or major paralysis, heart attack, coronary heart disease, angina, congestive heart failure or other heart disease), and ulcers and chronic inflamed bowel disorders.

#### Quality of life

The Short Form (SF)-12 is derived from the SF-36 instrument that is designed to measure generic quality of life [30]. The SF-12 has been validated across a number of chronic conditions and populations, including people with mental conditions and in Asian settings [31–33]. It generates scores across eight domains of health: physical function (PF), role-physical (RP), bodily pain (BP), general health (GH), vitality (VT), social function (SF), role-emotion (RE), and mental health (MH) which are then used to generate two summary scores - physical component summary (PCS) and mental component summary (MCS). PCS is calculated from PF, RP, BP and GH while MCS is obtained from VT, SF, RE and MH. Both summary scores range between 0 and 100, with higher scores indicating better health. In this study, the scores showed high internal consistency reliability for PCS (Cronbach’s alpha = 0.81) and MCS (Cronbach’s alpha = 0.82) domain scores.

#### Overall health

Self-reported overall health was obtained by asking an independent single item, “How would you rate your overall health?” with a response scale of ‘1’ Poor to ‘5’ Excellent.

### Statistical analysis

SPSS Complex Samples Version 24 software was used to conduct the analysis. Analyses were weighted to adjust for over sampling, non-response and post-stratified for age and ethnicity distributions between the survey sample and the Singapore resident population in 2014. A descriptive analysis using means for the continuous variables and frequency distribution for categorical variables was used to obtain the characteristics of the overall sample and those with and without lifetime mental disorders. Statistical normality of Total PMH was tested using both graphical (normal probability plot) and statistical procedures (Kolmogorov–Smirnov test). General linear regression models were used to assess differences in Total PMH between those with and without life-time mental disorders. Bivariate analyses were conducted with the respective mental disorder (Yes or No) as independent variable(s) and Total PMH as a continuous dependent variable. All multivariable analysis used these dependent and independent variables and were adjusted for the effect of sociodemographic characteristics: Age, Gender (Male, Female), Ethnicity (Chinese, Malay, Indian, Other), Marital status (Single, Married, Separated/Divorced, Widowed), Education level (Primary and below, Secondary, Junior College, Vocational, Diploma, University), Employment status (Unemployed, Economically inactive, Employed), and Monthly household income in Singapore dollars (SGD) (Below 2000, 2000–3999, 4000–5999, 6000–9999, 10,000 & above) and having any chronic physical condition (Yes or No). Mediation analyses were conducted using ordinary least squares path analysis to examine whether the relation between mental disorders and health outcomes (quality of life and overall health) was mediated by PMH using the PROCESS Macro for SPSS [34]. The models were composed of any lifetime mental disorder (Yes, No) as an independent variable, the health outcomes (overall health and quality of life) as dependent variables, and Total PMH as the mediating variable. Mediation hypotheses were tested using the bias-corrected bootstrap method with 5000 samples to calculate confidence intervals (95%). Indirect effect (IE) was considered significant when the confidence interval did not contain zero [35].

## Results

Table 1 presents the sociodemographic characteristics of the sample. The mean age of the respondents who completed the PMH questionnaire was 42.1 years with most aged 18–34 years (34.9%). There were slightly more females (52.1%) than males (47.9%) with a higher proportion of Chinese (77.5%), married (55.7%), university educated (39.9%), employed (74.3%) and having a household income of 10,000 and above (25.5%) per month.

**Table 1:** Sociodemographic characteristics of the sample (*n* = 2270)

		n	%
**Age groups (Years)**	18–34	679	34.9
	35–49	618	33.3
	50–64	611	24.5
	65+	362	7.2
**Gender**	Female	1162	52.1
	Male	1108	47.9
**Ethnicity**	Chinese	701	77.5
	Malay	603	10.4
	Indian	693	8.1
	Other	273	4.0
**Marital status**	Single	666	37.9
	Separated/Divorced	106	4.9
	Widowed	79	1.5
	Married	1419	55.7
**Education level**	Primary and below	176	4.6
	Secondary	590	20.2
	Junior College	154	7.5
	Vocational	160	5.5
	Diploma	461	22.4
	University	728	39.9
**Employment**	Unemployed	114	5.1
	Economically inactive	585	20.6
	Employed	1571	74.3
**Monthly household income (in SGD)**	2000–3999	496	18.2
	4000–5999	424	20.0
	6000–9999	447	25.2
	10,000 & above	421	25.5
	< 2000	315	11.2

In this sample, the lifetime prevalence of any mental disorders was 14.8% (Table 2). The mean Total PMH in the overall sample was 4.46 (SD 0.48). Individuals with a lifetime history of any mental disorder had lower Total PMH (Table 2). Specifically, respondents with MDD, bipolar disorder, GAD and OCD had significantly lower Total PMH compared with individuals without these conditions. These findings remained significant after adjusting for respondents’ sociodemographic background and history of any chronic physical conditions (Multivariable analysis, Table 2).

**Table 2:** Association of lifetime mental disorders with Total Positive Mental Health

	Distribution		Total Positive Mental Health		Bivariate analysis				Multivariable analysis^**a**^			
	*n*	%	Mean	SD	β	95% CI		*P*	β	95% CI		*P*
						Lower	Upper			Lower	Upper	
**Any mental disorder**												
Yes	319	14.8	4.23	0.66	−0.265	−0.375	− 0.154	**< 0.001**	− 0.233	− 0.337	− 0.129	**< 0.001**
No	1951	85.2	4.50	0.67	REF				REF			
**Major depressive disorder**												
Yes	131	6.6	4.22	0.60	−0.257	−0.409	− 0.104	**0.001**	− 0.232	− 0.376	− 0.088	**0.002**
No	2139	93.4	4.48	0.68	REF				REF			
**Bipolar disorder**												
Yes	32	1.8	3.92	0.77	−0.553	−0.968	−0.138	**0.009**	−0.414	−0.755	− 0.073	**0.017**
No	2238	98.2	4.47	0.67	REF				REF			
**Generalised anxiety disorder**												
Yes	47	2.1	4.12	0.67	−0.346	−0.609	−0.083	**0.0100**	−0.319	−0.557	− 0.082	**0.008**
No	2223	97.9	4.47	0.68	REF				REF			
**Obsessive Compulsive Disorder**												
Yes	92	4.4	4.05	0.70	−0.433	−0.634	−0.231	**< 0.001**	−0.354	− 0.526	− 0.183	**< 0.001**
No	2178	95.6	4.48	0.67	REF				REF			
**Alcohol use disorders**												
Yes	90	4.6	4.31	0.55	−0.154	−0.342	0.033	0.107	−0.086	− 0.274	0.102	0.371
No	2180	95.4	4.47	0.68	REF				REF			

^a^ General linear regression models adjusted for socio-demographic characteristics (as described in Table 1) and history of any chronic physical condition (Y/N)

Table 3 presents the associations of mental disorders with overall health and quality of life (MCS and PCS). Having any mental disorder was significantly and negatively associated with the health outcomes denoting poorer scores in this group.

**Table 3:** Association of Any mental disorder with self-rated overall health and quality of life domains

	Health outcomes’ score		Bivariate analysis				Multivariable analysis^**a**^			
Any mental disorder	Mean	SD	β	Lower	Upper	*P*	β	Lower	Upper	*P*
	Overall health									
Yes	3.24	0.62	−0.266	−0.409	− 0.124	**< 0.001**	− 0.352	−0.491	− 0.213	**< 0.001**
No	3.51	0.51	REF				REF			
	Quality of Life- Mental Component Summary									
Yes	51.5	5.12	−4.808	−6.039	−3.576	**< 0.001**	−4.485	−5.78	−3.191	**< 0.001**
No	56.3	6.37	REF				REF			
	Quality of Life- Physical Component Summary									
Yes	54.1	5.97	−0.332	−1.229	0.565	0.468	−1.019	−1.947	− 0.091	**0.031**
No	54.4	5.23	REF				REF			

^a^ General linear regression models adjusted for socio-demographic characteristics (as described in Table 1) and history of any chronic physical condition (Y/N)

**Fig. 1:**
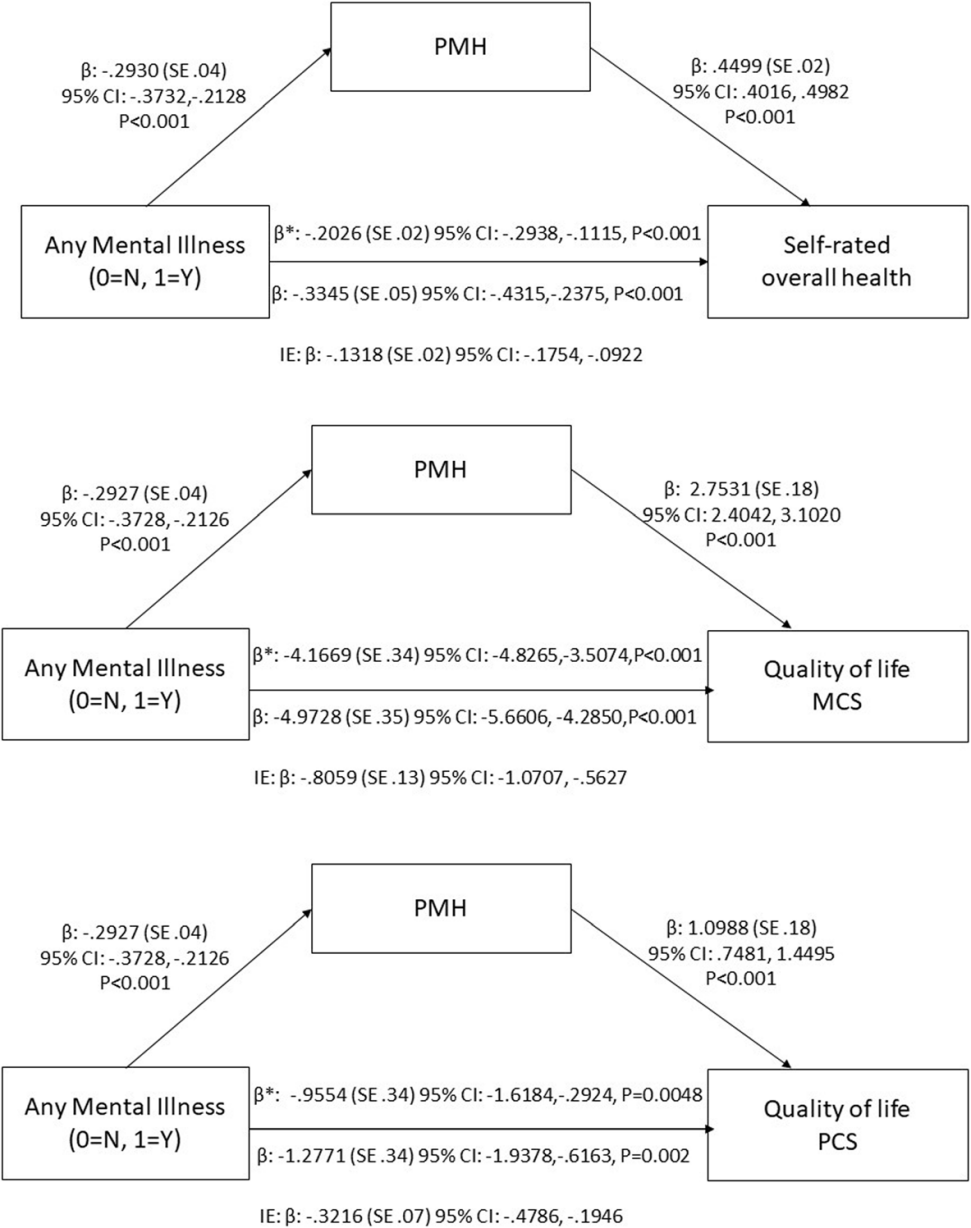
Mediation effect of PMH on the relationship between mental disorders and self-rated overall health and quality of life domains

Figure 1 presents results of the path analyses that tested mediating effect of Total PMH on the relationships between lifetime mental disorders and health outcomes. Total PMH fully mediated the relationships between mental disorder and overall health (IE size: −.1318), MCS (IE size: −.8059) and PCS (IE size: −.3216). This shows that higher levels of Total PMH in individuals with mental disorders reduced the effect sizes of the associations between mental disorders and health outcomes.

## Discussion

Knowledge about the state, correlates and role of PMH is important for informing mental health promotion and intervention programmes. This study estimated the level of PMH in a large general population sample comprising people with and without mental disorders. Additionally, people with mental disorders had lower PMH and this association remained strong after adjusting for sociodemographic background and history of chronic physical conditions.

The study showed individuals meeting the criteria for a mental health disorder, in particular mood and anxiety disorders, had lower PMH than those without. Similar results were found in an earlier study among patients with depression, anxiety and schizophrenia in Singapore [11]. Of the mental disorders studied, lifetime mood (MDD and bipolar disorder) and anxiety (GAD and OCD) disorders showed significant associations with PMH even after controlling for important confounders such as history of chronic physical illnesses. These findings were expected given the preliminary studies in Singapore and the UK that have found association of PMH and mental well-being with either diagnosed mental disorders or symptoms of depression and anxiety [26, 36]. However, this is the first study that assessed PMH among individuals meeting DSM-IV criteria for AUD. Although the level of PMH was lower among people with AUD, this association did not meet statistical significance. Two previous studies conducted in large samples have generated inconclusive results in relation to alcohol consumption. While alcohol use was linked to lower mental wellbeing in the UK general population [37] and among students in New Zealand [38], the latter study found that heavy drinking was correlated with high self-esteem in males and low self-esteem in females. The study conducted in New Zealand also reported higher self-efficacy in males and females who drank alcohol. Although these studies did not conduct a diagnostic evaluation of AUD, the relationship between AUD and PMH needs further investigation.

An important consideration while interpreting the study results is the clinical relevance of the findings. Currently, the PMHI can provide information on PMH in the form of a total score which can be used for comparisons between individuals, populations and periods. However, a cut-off value for better or poorer PMH is not available. Total PMH scores obtained for individuals with mental disorders included in this study were much lower than the Singapore’s general population norm (mean Total PMH score: 4.61) [25], but slightly higher than that in mental health users (mean Total PMH score: 3.93) [26]. The severity of the illness or it’s symptoms could be a possible confounder of this association, which may limit clinically meaningful interpretations to be drawn from the results. The CIDI provides information on severity of illness by disorder-specific role impairments in four domains – home management, social life, work/school and ability to form and maintain close relationships [28]. However, we were unable to investigate this association given the lower representation of individuals with severe disorders in our sample. The influence of disorder severity should, therefore, be explored in future research investigating the relationship between PMH and mental disorders.

The impact of mental disorders on overall health and quality of life is well known [4]. Depression alone and in combination with chronic diseases is shown to produce the “greatest decrement in health” [39]. Mediation analysis conducted in this study identified that PMH significantly influenced health outcomes related to mental disorders. Although the concept of flourishing and the belief that mental health is distinct yet correlated with mental illnesses are widely accepted [13], to our knowledge, there are no previous studies assessing mediation of the association between mental disorders and related health outcomes by PMH. The data from this large national study thus provides evidence to support role of PMH as a distinct heath determinant over mental illness, and highlights its relevance in improving health outcomes in clinical populations.

This being the first study to assess mediation by PMH in mental disorders, it is difficult to draw direct comparisons with previous research on its mechanisms. However, there could be several possible explanations. In this study, PMH was assessed with the PMHI which provides a total score based on six different dimensions. One or more of these dimensions could impact health outcomes in people with mental disorders. For example, one of the dimensions assessed by the PMHI is emotional support. Increased contact with family members is found to lead to “positive and longstanding effect” on subjective quality of life development in people with psychosis [40]. In relation to the general coping dimension in the PMHI, adaptive coping strategies have shown to exert independent and positive effect over symptom levels and degree of stress, and influence perceived problem-solving efficacy [41]. Further research on the independent contribution of these dimensions could fully explain mediation by PMH.

Studies conducted elsewhere have indicated the likely mechanisms with which positive emotions may influence cognitive aspects of mood and anxiety for example, by “disengagement from negative stimuli” to perception of pleasant experience [42, 43]. This also helps in regulating negative emotions [44] leading to overall positive appraisal of self-rated outcomes such as life satisfaction and quality of life [45]. Besides the direct and indirect psychological impact of PMH, it may also act via biological mechanisms. Amygdala activity interacts with anxiety and depression to enhance or reduce emotion processing which could be affected in mental disorders [46]. A study on happiness which reflects subjective well-being showed that happier participants had greater amygdala responses to positive stimuli [47]. Thus, PMH via subjective well-being and positive appraisals may stimulate neural activity to improve health outcomes in people with mental disorders. Besides the biological and psychological mechanisms, health behaviours such as physical activity, are known to improve mood states and increase mental well-being [37, 48]. The interaction of such health behaviours with PMH should be considered in the assessment of health outcomes of people with mental disorders in future.

Taking into account results of the mediation models, PMH improvement interventions would be beneficial to promote quality of life in people with mental disorders. In a broader sense, this study further endorses the now well-accepted view that there is a need to move beyond just a focus on mental illness and incorporate measures on PMH in clinical practice. It is interesting to note that although mental health promotion has undergone a remarkable transformation, the ways of assessing the impact of these interventions is still largely illness-focused. A review of population-based mental health promotion interventions showed that although a multitude of functional, social, and cognitive measures were being employed, most interventions aimed to prevent a specific mental illness or symptoms (depression, anxiety, burnout, or stress) [49, 50]. For example, educational courses for adults that aim to prevent depression symptomatology [51], online mental health promotion and prevention interventions using computerized cognitive behavioural and resilience-focused therapy that have been effective in promoting youth well-being and reducing mental health problems such as anxiety and depression symptoms [52, 53], and media campaigns challenging mental health stigma and promoting positive attitude that have improved attitudes to treatment [54] hold promise and need further research in relation to PMH.

There is also a growing body of work in the area of PMH promotion. Positive psychology which acts towards improving mental well-being by building positive experiences, strengths and resources [55], has been shown to reduce depressive symptoms and increasing well-being among people with mental disorders [56]. Psychotherapy interventions based on positive psychology have proven effective in improving mental well-being in people with schizophrenia, depression and anxiety [56–58]. Given the high healthcare burden, there is also focus on self-management of mental health. A large network analysis conducted to assess the relationship between psychological distress and mental well-being in UK indicated that interventions that promote positive self-perception and positive mood can prove beneficial to psychological well-being and recommends enhancing self-esteem, self-confidence and cheerfulness to elevate public mental health [59]. However, research and evidence in the area of PMH is as yet insufficient. This study contributes to the limited literature and sheds light on the role of PMH and its possible impact on the lives of people with mental disorders.

The study, however, has several limitations. The cross-sectional study design does not allow making causal inferences. In this study, PMH was assessed as a composite total score. The PMHI can provide scores for six dimensions of PMH - general coping, emotional support, spirituality, interpersonal skills, personal growth and autonomy, and global affect. Detailed analyses for the independent dimensions were beyond the scope of this study. However, further analysis to assess the relative relevance of these PMH dimensions is necessary to identify specific components of PMH that contribute to improved health outcomes in people with mental disorders. Longitudinal studies that incorporate repeated measures to assess the fluctuations in PMH and health outcomes can also help fully understand the role of PMH. Although validated measures were used for assessing PMH, mental disorders and other health outcomes, this study relied on self-report that could lead to some bias in the findings. Due to the likely respondent burden, only select disorders were included in the survey and we were unable to assess association of PMH with other important mental disorders such as schizophrenia, neurocognitive disorders, and personality disorders. The response rate for this study was 30%, which could have resulted in selection bias in the findings. Use of objective measures for estimating overall health and quality of life would benefit future research. Finally, there are several definitions of PMH in the literature that have resulted in multiple measures that represent varied constructs of mental health and well-being. In this study, Total PMH represented six dimensions of mental health that were found to be relevant and valid in the Singapore population. However, it is important to consider cross-cultural appropriateness of these dimensions while applying the results to other populations.

## Conclusions

This study estimated levels of Total PMH among people with and without mental disorders, specifically MDD, bipolar disorder, GAD, OCD and AUD (abuse and dependence) in a large representative multi-ethnic sample. Higher levels of PMH seem to reduce the strength of the inverse associations between mental disorder and health outcomes, in terms of self-rated overall health and quality of life. Results support the notion that clinical interventions should incorporate approaches to improve PMH in routine practice. Although mental health promotion is largely devoted to prevention and treatment of mental illness, continued efforts are essential to improve PMH in people with mental disorders and monitor the relationship between these disorders and important health outcomes.

## Data Availability

The dataset generated and/or analysed during the current study are not publicly available due to funding restrictions but are available from the corresponding author on reasonable request.

## Abbreviations

AUD: Alcohol use disorders
CIDI 3.0: Composite International Diagnostic Interview version 3.0
DSM-IV: Diagnostic and Statistical Manual of Mental Disorders, 4th Edition
GAD: Generalised anxiety disorder
MCS: Mental component summary
MDD: Major depressive disorder
OCD: Obsessive compulsive disorder
PCS: Physical component summary
PMH: Positive mental health
PMHI: Positive Mental Health Instrument
SF-12: Short Form-12
SMHS: Singapore Mental Health Study
UK: United Kingdom
WHO: World Health Organization

## References

[1] Department of Statistics Singapore (2018). Population Trends.

[2] Chong SA, Abdin E, Vaingankar JA, Heng D, Sherbourne C, Yap M (2012). A population-based survey of mental disorders in Singapore. Ann Acad Med Singap.

[3] Subramaniam M, Abdin E, Vaingankar JA, Shafie S, Chua BY, Sambasivam R (2019). Tracking the mental health of a nation: prevalence and correlates of mental disorders in the second Singapore mental health study. Epidemiol Psychiatr Sci.

[4] Whiteford HA, Ferrari AJ, Degenhardt L, Feigin V, Vos T (2015). The global burden of mental, neurological and substance use disorders: an analysis from the global burden of disease study 2010. PLoS One.

[5] Walker ER, McGee RE, Druss BG (2015). Mortality in mental disorders and global disease burden implications: a systematic review and meta-analysis. JAMA Psychiatry.

[6] Saarni SI, Suvisaari J, Sintonen H, Pirkola S, Koskinen S, Aromaa A (2007). Impact of psychiatric disorders on health-related quality of life: general population survey. Br J Psychiatry.

[7] Steel Z, Marnane C, Iranpour C, Chey T, Jackson JW, Patel V (2014). The global prevalence of common mental disorders: a systematic review and meta-analysis 1980-2013. Int J Epidemiol.

[8] Vigo Daniel V, Kestel Devora, Pendakur Krishna, Thornicroft Graham, Atun Rifat (2019). Disease burden and government spending on mental, neurological, and substance use disorders, and self-harm: cross-sectional, ecological study of health system response in the Americas. The Lancet Public Health.

[9] Phua HP, Chua AV, Ma S, Heng D, Chew SK (2009). Singapore's burden of disease and injury 2004. Singap Med J.

[10] Lim D, Lee WK, Park H (2016). Disability-adjusted life years (DALYs) for mental and substance use disorders in the Korean burden of disease study 2012. J Korean Med Sci.

[11] World Health Organization (2005). Promoting mental health: Concepts, emerging evidence, practice.

[12] Jahoda M (1958). Current concepts of positive mental health. Joint commission on mental health and illness monograph series.

[13] Keyes CLM (2002). The mental health continuum: from languishing to flourishing in life. J Health Soc Behav.

[14] Gilmour H (2014). Positive mental health and mental illness. Health Rep.

[15] Keyes CLM, Eisenberg D, Perry GS, Dube SR, Kroenke K, Dhingra SS (2012). The relationship of level of positive mental health with current mental disorders in predicting suicidal behavior and academic impairment in college students. J Am Coll Heal.

[16] Seow LS, Vaingankar JA, Abdin E, Sambasivam R, Jeyagurunathan A, Pang S (2015). Positive mental health in outpatients with affective disorders: associations with life satisfaction and general functioning. J Affect Disord.

[17] Teismann T, Brailovskaia J, Totzeck C, Wannemüller A, Margraf J (2018). Predictors of remission from panic disorder, agoraphobia and specific phobia in outpatients receiving exposure therapy: the importance of positive mental health. Behav Res Ther.

[18] Haque A (2010). Mental health concepts in Southeast Asia: diagnostic considerations and treatment implications. Psychol Health Med.

[19] Hernandez M, Nesman T, Mowery D, Acevedo-Polakovich ID, Callejas LM (2009). Cultural competence: a literature review and conceptual model for mental health services. Psychiatr Serv.

[20] Christopher JC (1999). Situating psychological well-being: exploring the cultural roots of its theory and research. J Couns Dev.

[21] Suh EM (2002). Culture, identity consistency, and subjective well-being. J Pers Soc Psychol.

[22] Vaingankar JA, Subramaniam M, Chong SA, Abdin E, Orlando Edelen M, Picco L (2011). The positive mental health instrument: development and validation of a culturally relevant scale in a multi-ethnic Asian population. Health Qual Life Outcomes.

[23] Vaingankar JA, Subramaniam M, Lim YW, Sherbourne C, Luo N, Ryan G (2012). From well-being to positive mental health: conceptualization and qualitative development of an instrument in Singapore. Qual Life Res.

[24] Vaingankar JA, Subramaniam M, Abdin E, Picco L, Phua A, Chua BY (2013). Socio-demographic correlates of positive mental health and differences by depression and anxiety in an Asian community sample. Ann Acad Med Singap.

[25] Vaingankar JA, Subramaniam M, Tan LWL, Abdin E, Lim WY, Wee HL (2018). Psychometric properties and population norms of the positive mental health instrument in a representative multi-ethnic Asian population. BMC Med Res Methodol.

[26] Sambasivam R, Vaingankar JA, Chong SA, Abdin E, Jeyagurunathan A, Seow LS (2016). Positive mental health in outpatients: comparison within diagnostic groups. BMC Psychiatry.

[27] Jeyagurunathan A, Vaingankar JA, Abdin E, Sambasivam R, Seow E, Pang S (2017). Gender differences in positive mental health among individuals with schizophrenia. Compr Psychiatry.

[28] Kessler RC, Üstün TB (2004). The world mental health (WMH) survey initiative version of the world health organization (WHO) composite international diagnostic interview (CIDI). Int J Methods Psychiatr Res.

[29] Haro JM, Arbabzadeh-Bouchez S, Brugha TS, de Girolamo G, Guyer ME, Jin R, Lepine JP, Mazzi F, Reneses B, Vilagut G, Sampson NA, Kessler RC (2006). Concordance of the composite international diagnostic interview version 3.0 (CIDI 3.0) with standardized clinical assessments in the WHO world mental health surveys. Int J Methods Psychiatr Res.

[30] Ware J, Kosinski M, Keller SD (1996). A 12-item short-form health survey: construction of scales and preliminary tests of reliability and validity. Med Care.

[31] Huo T, Guo Y, Shenkman E, Muller K (2018). Assessing the reliability of the short form 12 (SF-12) health survey in adults with mental health conditions: a report from the wellness incentive and navigation (WIN) study. Health Qual Life Outcomes.

[32] Salyers MP, Bosworth HB, Swanson JW, Lamb-Pagone J, Osher FC (2000). Reliability and validity of the SF-12 health survey among people with severe mental illness. Med Care.

[33] Brazier J, Connell J, Papaioannou D (2014). A systematic review, psychometric analysis and qualitative assessment of generic preference-based measures of health in mental health populations and the estimation of mapping functions from widely used specific measures. Health Technol Assess.

[34] Hayes AF (2013). Introduction to mediation, moderation, and conditional Process analysis.

[35] Hayes Andrew F., Rockwood Nicholas J. (2017). Regression-based statistical mediation and moderation analysis in clinical research: Observations, recommendations, and implementation. Behaviour Research and Therapy.

[36] Pitchforth Jacqueline, Fahy Katie, Ford Tamsin, Wolpert Miranda, Viner Russell M., Hargreaves Dougal S. (2018). Mental health and well-being trends among children and young people in the UK, 1995–2014: analysis of repeated cross-sectional national health surveys. Psychological Medicine.

[37] Stranges S, Samaraweera PC, Taggart F, Kandala NB, Stewart-Brown S (2014). Major health-related behaviours and mental well-being in the general population: the health survey for England. BMJ Open.

[38] Blank ML, Connor J, Gray A, Tustin K (2016). Alcohol use, mental well-being, self-esteem and general self-efficacy among final-year university students. Soc Psychiatry Psychiatr Epidemiol.

[39] Moussavi S, Chatterji S, Verdes E, Tandon A, Patel V, Ustun B (2007). Depression, chronic diseases, and decrements in health: results from the world health surveys. Lancet.

[40] Gardsjord ES, Romm KL, Friis S, Barder HE, Evensen J, Haahr U (2016). Subjective quality of life in first-episode psychosis. A ten-year follow-up study. Schizophr Res.

[41] Aldwin CM, Revenson TA (1987). Does coping help? A re-examination of the relation between coping and mental health. J Pers Soc Psychol.

[42] Joormann J, Stanton CH (2016). Examining emotion regulation in depression: a review and future directions. Behav Res Ther.

[43] Garland EL, Fredrickson B, Kring AM, Johnson DP, Meyer PS, Penn DL (2010). Upward spirals of positive emotions counter downward spirals of negativity: insights from the broaden-and-build theory and affective neuroscience on the treatment of emotion dysfunctions and deficits in psychopathology. Clin Psychol Rev.

[44] Tugade MM, Fredricksson BL (2004). Resilient individuals use positive emotions to bounce back from negative emotional experiences. J Pers Soc Psychol.

[45] Gross JJ, John OP (2003). Individual differences in two emotion regulation processes: implications for affect, relationships, and well-being. J Pers Soc Psychol.

[46] Greenberg T, Bertocci MA, Chase HW, Stiffler R, Aslam HA, Graur S (2017). Mediation by anxiety of the relationship between amygdala activity during emotion processing and poor quality of life in young adults. Transl Psychiatry.

[47] Cunningham WA, Kirkland T (2013). The joyful, yet balanced, amygdala: moderated responses to positive but not negative stimuli in trait happiness. Soc Cogn Affect Neurosci.

[48] Fox KR (1999). The influence of physical activity on mental well-being. Public Health Nutr.

[49] Enns J, Holmqvist M, Wener P, Halas G, Rothney J, Schultz A (2016). Mapping interventions that promote mental health in the general population: a scoping review of reviews. Prev Med.

[50] Jane’-Llopis E, Barry M, Hosman C, Patel V (2005). Mental health promotion works:a review. Promot Educ.

[51] Allart-van Dam E, Hosman C, Hoogduin C, Schaap C (2003). Short term and mediating results of the coping with depression course as indicated prevention: a randomized controlled trial. Behav Ther.

[52] Clarke AM, Kuosmanen T, Barry MM (2015). A systematic review of online youth mental health promotion and prevention interventions. J Youth Adolesc.

[53] Dray J, Bowman J, Campbell E, Freund M, Wolfenden L, Hodder RK (2017). Systematic review of universal resilience-focused interventions targeting child and adolescent mental health in the school setting. J Am Acad Child Adolesc Psychiatry.

[54] Sartorius N (1997). Fighting schizophrenia and its stigma. A new World Psychiatric Association educational programme. Br J Psychiatry.

[55] Seligman M, Steen T, Park N, Peterson C (2005). Positive psychology progress: empirical validation of interventions. Am Psychol.

[56] Bolier L, Haverman M, Westerhof G, Riper H, Smit F, Bohlmeijer E (2013). Positive psychology interventions: a meta-analysis of randomized controlled studies. BMC Public Health.

[57] Schrank B, Brownell T, Jakaite Z, Larkin C, Pesola F, Riches S (2016). Evaluation of a positive psychotherapy group intervention for people with psychosis: pilot randomised controlled trial. Epidemiol Psychiatr Sci.

[58] Trompetter HR, Lamers SMA, Westerhof GJ, Fledderus M, Bohlmeijer ET (2017). Both positive mental health and psychopathology should be monitored in psychotherapy: confirmation for the dual-factor model in acceptance and commitment therapy. Behav Res Ther.

[59] Stochl J, Soneson E, Wagner AP, Khandaker GM, Goodyer I, Jones PB (2018). Identifying key targets for interventions to improve psychological wellbeing: replicable results from four UK cohorts. Psychol Med.

